# Supplementary figures and images for: In-hospital versus after-discharge complete revascularization in patients with ST segment elevation myocardial infarction and multivessel disease. REVIVA-ST trial
Source: PLoS One. 2024 May 14;19(5):e0303284. doi: 10.1371/journal.pone.0303284 (PMC11093342; doi:10.1371/journal.pone.0303284)

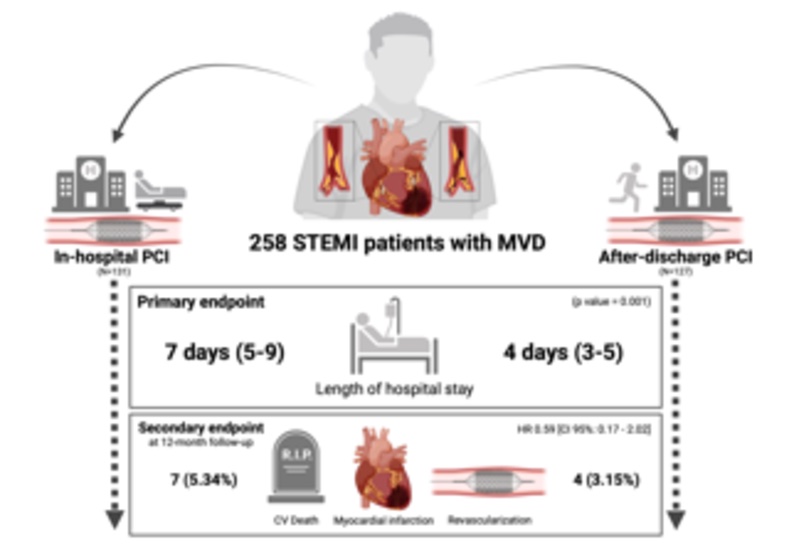

Supplement: S1 Graphical abstract — (JPG) [file pone.0303284.s002.jpg]
