# Supplementary material for: In-hospital versus after-discharge complete revascularization in patients with ST segment elevation myocardial infarction and multivessel disease. REVIVA-ST trial
Source: PLoS One. 2024 May 14;19(5):e0303284. doi: 10.1371/journal.pone.0303284 (PMC11093342; doi:10.1371/journal.pone.0303284)
Supplement: S1 File — (DOCX) [file pone.0303284.s003.docx]

**In-hospital versus after-discharge complete revascularization in patients with ST segment elevation myocardial infarction and multivessel disease.**

**REVIVA-ST trial.**

Eva Rumiz, MD, PhD.

Cardiology Department, Consorcio Hospital General Universitario de Valencia.

1.Introduction 3

2. Study design 7

2.2 Hypothesis 7

2.3 Primary outcome 7

2.4 Secondary outcomes 7

3. Methods 8

3.1 Study population 8

3.2 Inclusion criteria 8

3.3 Exclusion criteria 9

4. Study procedure 9

4.1 Informed consent 9

4.2 Study treatment........................................................................................................10

4.2.1 In-hospital complete revaacularization 10

4.2.2 After-discharge comnplete revascularization 11

5. Sample size calculation and statistical analysis…….....…………………............……………...11

5.1 Sample size calculation 11

5.2 Stadistical analysis 11

6. Ethical, deontological and regulatory considerations 12

7. References ………………………..……………………………………………………….…………………………13

**1.INTRODUCTION**

Primary angioplasty (PA) is the reperfusion therapy of choice in ST-segment elevation myocardial infarction (STEMI)^1,2^. Several studies have shown a significant reduction in major adverse cardiac events (MACE) compared to fibrinolysis as a reperfusion strategy^3,4^.

About 40-50% of patients with STEMI have multivessel disease (MVD)^5^, defined as at least one angiographic stenosis greater than or equal to 50-70% in a vessel larger than 2 mm in diameter other than the vessel responsible for the STEMI and amenable to revascularization. These patients constitute a high-risk subgroup with a worse short- and long-term prognosis compared to patients with single-vessel disease^6^.

The 2012 European Society of Cardiology clinical practice guidelines for the management of STEMI patients, establish percutaneous coronary intervention (PCI) limited to the culprit vessel during PA with a class IIa recommendation, with multivessel revascularization only considered in case of haemodynamic instability^7^. This indication is mainly based on retrospective studies^8^ and meta-analyses^9^ where complete revascularization (CR) during PA was associated with increased mortality compared to both a conservative strategy of revascularization of the culprit vessel only and a more aggressive strategy of deferred CR of the non-culprit stenosis. The latter strategy of delayed multivessel revascularization has been associated with the greatest reduction in MACE during follow-up.

In recent years, 4 clinical trials PRAMI^10^, CULPRIT^11^, DANAMI 3 PRIMULTI^12^ and COMPARE-ACUTE^13^ have been published, comparing a strategy of revascularization of the culprit vessel only with a strategy of preventive CR or guided by analysis of fractional flow reserve (FFR).

The PRAMI study enrolled 465 patients with MVO, defined as the presence of angiographic stenosis ≥ 50% in non-culprit vessel. They were randomised 1:1 to PA of the culprit vessel and medical treatment of the remaining stenosis vs. percutaneous treatment of all stenoses during PA. This study was stopped early because of a significant difference in the primary endpoint (death, infarction, need for repeat revascularization) in favour of CR. A 65% relative reduction in the risk of the combined event was observed mainly due to a reduction in the incidence of angina and/or infarction, and need for repeat revascularization. However, no differences in total mortality during follow-up were observed between the two groups.

Later, the CULPRIT study compared the same revascularization strategies as the PRAMI study, however the presence of angiographic stenosis ≥ 70% in a non-culprit vessel was considered MVO. The study included 296 patients randomised 1:1 to both strategies, with the timing of CR left to the operator's discretion; CR was deferred to a second procedure in 27% of patients. Similar to the PRAMI study, a benefit of CR was observed with a significant reduction in the primary endpoint (death, myocardial infarction, heart failure, and need for repeat revascularization). However, although there is a trend towards a lower incidence of each of the in-hospital events, statistical significance was not reached for any of them separately.

Later the DANAMI-3 PRIMULTI study and recently the COMPARE ACUTE study found that FFR-guided CR was associated with a significant reduction in the risk of MACE compared to revascularization of only the culprit vessel.

These results prompted a modification of the American Society of Cardiology guidelines in 2015 from a class III recommendation to IIb for revascularization of non-culprit lesions during PA in haemodynamically stable patients^14^. Similarly, the recent European Society of Cardiology guidelines for the management of ST-elevation acute myocardial infarction establish a class IIa recommendation for routine revascularization of non-culprit lesions before hospital discharge, without establishing the optimal time for this to be performed.

All the aforementioned studies compare a strategy of CR with a strategy of treatment of only the culprit vessel and medical treatment of the remaining stenosis. In all of them, a significant reduction of the combined target was observed, mainly due to a decrease in the need for repeat revascularization. However, to date, there is no randomised study comparing two complete revascularization strategies performed at different times, with only retrospective studies of small size available. Politi et al^16^ randomised 214 patients to three different revascularization strategies; immediate revascularization during PA, delayed revascularization after hospital discharge and revascularization limited to the culprit vessel. A higher incidence of MACE was observed in the more conservative strategy limited to PCI of the culprit vessel (50%) compared to 20% and 23% in the other 2 CR strategies. This reduction in the risk of MACE with the preventive revascularization strategies was maintained in multivariate analysis after adjustment for multiple variables, with a risk reduction of 63% and 60%, respectively, with no differences between the two CR strategies.

In a retrospective study conducted at our centre, 807 patients undergoing PA from January 2009 to December 2015 were analysed. 398 (49.3%) patients showed MVD on coronary angiography, considered as the existence of at least one angiographic stenosis ≥70% in non-culprit vessel, of which 187 underwent only revascularization of the culprit vessel and 184 underwent preventive CR, 45 of them during PA. Of the 139 patients undergoing deferred CR, 69 (49.6%) were performed during the index admission and 70 (50.4%) in a second procedure on an outpatient basis after hospital discharge. During a median follow-up of 10.5 months (interquartile range: 5-30) no significant differences in all-cause mortality, cardiac death, reinfarction or readmission for heart failure were observed. However, an increase in total days of hospital stay was observed in patients undergoing complete revascularization during the index admission (8.2 ± 4 vs. 6.5 ± 2.9; p<0.001).

In the recent clinical practice guidelines of the European Society of Cardiology, on the management of patients with STEMI, for the first time establishes a class of recommendation for early discharge (48-72 hours) of low-risk patients undergoing PA. The PAMI II^17^ criteria for identifying low-risk patients include age <75 years, left ventricular ejection fraction greater than 45%, successful PCI and one- or two-vessel disease, so the existence of stenosis in non-culprit vessels is not a priori a high-risk criterion. However, a strategy of complete in-hospital revascularization could lead to an increase in the total length of stay of our patients, preventing early discharge as recommended by current clinical practice guidelines.

**2. STUDY DESIGN**

2.1. SUMMARY OF THE STUDY DESIGN

This is a prospective randomised single centre study. The study will be conducted in accordance with the principles set out in the Declaration of Helsinki (1996) and the International Conference on Harmonisation of Good Clinical Practice.

2.2 HYPOTHESIS

Complete outpatient revascularization of non-culprit lesions in patients with multivessel disease undergoing primary angioplasty significantly reduces hospital stay compared to in-hospital revascularization for the index event and is at least as safe a strategy.

2.3 PRIMARY OUTCOME

1º. To determine a reduction in hospital stay among patients undergoing complete revascularization during the index admission and on an outpatient basis.

2.4 SECONDARY OUTCOMES

1º. To determine the non-inferiority of the strategy of complete outpatient revascularization in reducing the combined safety event (death, reinfarction, need for repeat revascularization) at 6 and 12 months follow-up.

To assess the presence of ischaemia by analysing the fractional flow reserve of angiographically moderate stenoses (≥50% and <70%) in proximal segments of the anterior descending, circumflex or right coronary artery.

**3. METHODS**

3.1 STUDY POPULATION

Patients aged 18-90 years admitted for STEMI whose reperfusion strategy is PA and who show MVD on coronary angiography, considered as the presence of at least one stenosis ≥ 70% in non-culprit vessel greater than 2 mm in diameter or presence of stenosis ≥ 50% in proximal segment of non-culprit vessel, quantified by QCA, will be prospectively included. Non-culprit stenoses are those identified by the operator as not responsible for STEMI. All patients must meet all inclusion criteria and no exclusion criteria.

3.2 INCLUSION CRITERIA

Candidates shall be those patients who meet all of the following conditions:

1) Patients aged between 18 and 90 years.

2) Presentation of STEMI of less than 12 hours of evolution and candidate for primary angioplasty.

3). Presence of at least ≥ 70% angiographic stenosis in non-culprit vessel > 2mm in diameter, or ≥ 50% in proximal segment of non-culprit vessel measured by QCA.

4). The participant is willing and able to give informed consent for participation in the study.

3.3 EXCLUSION CRITERIA

Candidates must not meet any of the following conditions:

1) Significant left main coronary artery disease.

2) STEMI due to stent thrombosis.

3) Chronic total occlusion of non-culprit vessel.

4) Severe non-culprit vessel stenosis with distal TIMI <3.

5) Non-culprit vessel stenosis not amenable to percutaneous revascularization.

6) Presence of significant valvular heart disease requiring surgery.

7) Killip class IV at the time of presentation.

8) Inability to obtain informed consent.

**4. STUDY PROCEDURE**

4.1 INFORMED CONSENT

The participant should sign the informed consent form before any specific trial procedure is performed.

The written version of the consent and information sheet should be presented to the patient candidate and should include the following information: the exact nature of the trial, the implications and limitations of the protocol, and the risks inherent to such participation. This document should detail that the participant is free to withdraw from the study at any time and for any reason and without prejudice to future care, and without the obligation to give the reason for withdrawal.

The patient shall be allowed as much time as necessary to examine the information and the possibility to question the investigator, his or her general practitioner or other independent parties to decide whether to participate in the study. Written informed consent is obtained by the signature and date of the participant and the person who submitted and obtained the informed consent. The person who obtained the consent must be suitably qualified and experienced, and must have been authorised to do so by the principal investigator. A copy of the signed informed consent shall be given to the participants. The original signed consent document should be retained at the trial site.

4.2 STUDY TREATMENT

Patients will be randomised in a 1:1 computerised fashion to in-hospital CR or after-discharge CR.

4.2.1 In-hospital complete revascularization

In-hospital CR shall be performed at least 24 hours after PA of the culprit vessel. Patients with angiographic stenoses assessed by QCA greater than or equal to 70% will undergo direct PCI and stent implantation, following current recommendations. Those stenoses angiographic lesions (≥ 50%-<70%), will be studied by pressure guidewire to assess their functionality. According to the established FFR values^8^, those lesions with an FFR of FFR≤0,80 will be revascularized.

Demographic, clinical and electrocardiographic variables will be collected on admission, as well as the usual analytical parameters at 24, 48 and 72 hours after STEMI, according to the clinical practice guidelines and the protocol of our department.

Follow-up will be performed at 1 month, 6 months and 1 year in the outpatient clinic and/or by telephone.

4.2.2 After-discharge complete revascularization

After-discharge CR will be performed 4-6 weeks after hospital discharge under optimal medical treatment. Patients with angiographic stenosis assessed by QCA greater than or equal to 70% will undergo direct angioplasty and stent implantation, following current recommendations. Those with moderate angiographic stenosis (≥ 50%-<70%) will be studied by pressure guidewire to assess their functionality. According to the established FFR values^8^, those lesions with an FFR of FFR≤0,80 will be revascularized.

Demographic, clinical and electrocardiographic variables will be collected on patient admission, as well as the usual analytical parameters at 24, 48 and 72 hours after the following current recommendations STEMI, according to the clinical practice guidelines and the protocol of our service.

Follow-up will be carried out at one month, six months and one year in the outpatient clinic and/or by telephone.

**5. SAMPLE SIZE CALCULATION AND STATISTICAL ANALYSIS.**

5.1 Sample size calculation

The calculation of the sample size was based on the mean length of stay between the two strategies obtained in retrospective studies^18-19^ using the Stata 13 "sampsi" programme. Assuming a statistical power of 95% and a significance level of 5%, the sample size for each group is estimated at 110 patients. Taking into account loss to follow-up, the sample size has been increased by 15%, so that 125 patients will be included in each strategy.

5.2 Statistical analysis

Quantitative variables will be expressed as mean ± 1 standard deviation, or as median (interquartile ranges) in the absence of Gaussian distribution. Discrete variables shall be expressed with the frequency distribution. Continuous variables shall be compared with Student's t-test. Discrete variables shall be compared with the X2 test. In all cases a p<0.05 will be considered significant. The statistical package STATA 13.1 will be used for data analysis.

**6. ETHICAL, DEONTOLOGICAL AND REGULATORY CONSIDERATIONS**

The investigator will ensure that this trial was conducted in accordance with the principles of the Declaration of Helsinki, ICH Good Clinical Practice guidelines and in full compliance with relevant regulations.

The protocol, informed consent form, patient information sheet and all applicable documents will be submitted to an Ethics Committee and written approval will be given by the Regulatory Authority.

All substantial modifications to the original approved documents will also be submitted to an Ethics Committee and written approval will be given by the Regulatory Authority.

Study personnel will ensure that the anonymity of participants is maintained. Participants will be identified only by a participant identification number in the data collection booklet and in any electronic database. All documents will be stored securely and will only be accessible by study staff and authorised personnel. The study will comply with data protection law, which requires data to be rendered anonymous as soon as it becomes mandatory to do so.

**7. REFERENCES**

1. Antman EM, Anbe DT, Armstrong PW, et al. ACC/AHA guidelines for the management of patients with ST-elevation myocardial infarction: a report of the American College of Cardiology/American Heart Association Task Force on Practice Guidelines (Committee to Revise the 1999 Guidelines for the Management of Patients With Acute Myocardial Infarction). J Am Coll Cardiol 2004; 44:E1–211.

2. Antman EM, Hand M, Armstrong PW, et al. 2007 focused update of the ACC/AHA 2004 guidelines for the management of patients with ST-elevation myocardial infarction: a report of the American College of Cardiology/American Heart Association Task Force on Practice Guidelines. J Am Coll Cardiol 2008;51:210 – 47.

3. Keeley EC, Boura JA, Grines CL. Primary angioplasty versus intravenous thrombolytic therapy for acute myocardial infarction: a quantitative review of 23 randomised trials. Lancet. 2003;361:13–20.

4. Andersen HR, Nielsen TT, Vesterlund T, et al. Danish multicenter randomized study on fibrinolytic therapy versus acute coronary angioplasty in acute myocardial infarction: rationale and design of the DANish trial in Acute Myocardial Infarction-2 (DANAMI-2). Am Heart J. 2003;146:234 – 41.

5. O´Gara PT, Kushner FG, Ascheim DD, et al. 2013 ACCF/AHA Guideline for the management of ST elevation myocardial infarction. J Am Coll Cardiol. 2013; 61:78-140.

6. Sorajja P, Gersh BJ, Cox DA, et al. Impact of multivessel disease on reperfusión success and clinical outcomes in patients undergoing primary percutaneous coronary intervention for acute myocardial infarction. Eur Heart Jour 2007;28:1709-1716.

7. ESC Guidelines for the management of acute myocardial infarction in patients presenting with ST-segment elevation . Eur heart J 2012;33:2569-2619.

8. Hannan E, Samadashvili Z, Walford G, et al. Culprit vessel percutaneous coronary intervention versus multivessel and staged percutaneous coronary intervention for ST-segment elevation myocardial infarction patients with multivessel disease. JACC Cardiovasc Interv 2010;3(1):22-31.

9. Vlaar PJ, Mahmoud KD, Holmes DR, et al. Culprit vessel only versus multivessel and staged percutaneous coronary intervention for multivessel disease in patients presenting with ST-segment elevation myocardial infarction. J Am Coll Cardiol 2011;58:692-703.

10. Wald DS, Morris JK, Wald NJ, et al. Randomized trial of preventive angioplasty in myocardial infarction. PRAMI Investigators. N Engl J Med 2013; 369:115-23.

11. Gershlick A, Khan JN, Kelly D, et al. Lesion only revascularization in patients undergoing primary percutaneous coronary intervention for STEMI and multivessel disease. J Am Coll Cardiol 2015;65:963-972.

12. Engstrom T, Kelbaek H, Helqvist S, et al. Complete revascularisation of the culprit lesión only in patients with ST-segment-elevation myocardial infarction and multivessel disease. (DANAMI-3—PRIMULTI): an open-label, randomised controlled trial Lancet 2015; 386:665-671.

13. Smits PC, Abdel-Wahab M, Neumann FJ, et al. Fractional flow reserve-guided multivessel disease angioplasty in myocardial infarction. N Engl J Med 2017;376:1234-1244.

14. Levine GN, Bates ER, Blankenship JC, et al. 2015 ACC/AHA/SCAI Focused update on primary percutaneous coronary intervention for patients with ST-elevation myocardial infarction. J Am Coll Cardiol. 2016;67(10):1235-1250.

15. Ibanez B, James S, Agewall S, et al. ESC Guidelines for the management of acute myocardial infarction in patients presenting with ST segment elevation myocardial infarction. Eur Heart J. 2017;00:1-66.

16. Politi L, Sgura F, Rossi R, et al. A randomised trial of target-vessel versus multi-vessel revascularisation in ST-elevation myocardial infarction: major adverse cardiac events during long-term follow up. Heart 2010;96:662-667.

17. Grines CL, Marsalese DL, Brodie B, et al. Safety and cost-effectiveness of early discharge after primary angioplasty in low risk patients with acute myocardial infarction. PAMI-II Investigators. Primary Angioplasty in Myocardial Infarction. J Am Coll Cardiol 1998;31: (5):967-972.

18.Russo JJ, Wells GA, Cheong AY, So DY, Glover CA, Froeschl MPV, et al. Safety and efficacy of staged percutaneous coronary intervention during index admission for ST-segment elevation myocardial infarction with multivessel coronary disease. Am J Cardiol 2015;116:1157-1162.

19.Ijsselmuiden AJJ, Ezechiels JP, Westendorp ICD, Tijssen JGP, Kiemeneji F, Slagboom T, et al. Complete versus culprit vessel percutaneous coronary intervention in multivessel disease: A randomized comparison. Am Heart J 2004;148:467-74.
