# Supplementary material for: In-hospital versus after-discharge complete revascularization in patients with ST segment elevation myocardial infarction and multivessel disease. REVIVA-ST trial
Source: PLoS One. 2024 May 14;19(5):e0303284. doi: 10.1371/journal.pone.0303284 (PMC11093342; doi:10.1371/journal.pone.0303284)
Supplement: Attachment — Submitted filename: Response to Reviewers.docx [file pone.0303284.s005.docx]

**Response to Reviewers**

**Ref.: PONE-D-23-18808**

We acknowledge to the Editorial Office and Reviewers for their comments and constructive suggestions, which undoubtedly have improved the quality of this manuscript.

**Reviewer #1:**

In this trial, the colleagues compared two different strategies of complete coronary revascularization in ACS STEMI patients, showing higher hospital stay in in hospital admission complete revascularization strategy and no difference in term of MACE.

The topic of the manuscript is interesting, however there are some issues to solve and revise:

1) There are too many typos in the text (some examples: trategies in the last sentence of introduction section, PressueWire, also a lot of times the authors write “de” instead of “the”). Please check all the typos and revise.

- Response: *Attending the reviewer’s comment, we have checked and revised all the typos present in the manuscript.*

2) The endpoints description should be written more fluently and not as a list. Please revise. Moreover, after double point (: ) the authors should not write in uppercase. Please revise.

- Response: *Attending the reviewer’s suggestion, the endpoints description has been written more fluently and not as a list, and after double point we have revised the text. Therefore, the Endpoints and follow-up paragraph (page 5) has been changed: “The primary endpoint was to evaluate the differences in length of hospital stay between treatment strategies. The secondary endpoints were: the composite of cardiovascular death, MI, or ischemia-driven revascularization during 12 months follow-up; as well as, to study the influence of the timing of revascularization on fractional flow reserve (FFR) measurements of intermediate nonculprit lesions located at proximal coronary segments”.*

3) The authors report no difference in intensive care unit stay between the two groups. Please specify if the intensive care stay in the staged revascularization group is related to first or second hospital admission ( and if it is related to the second admission, please clarify why elective patients treated for elective PCI should go in coronary intensive care unit).

-Response: *Answering the reviewer’s question, the intensive care stay in the staged revascularization group is only related to the first hospital admission (index admission). The after-discharge revascularization was always a programmed-elective procedure through radial artery access with no procedure-related complications, and therefore these patients needed no hospital stay in that second admission, being all discharged home in the same day of the procedure.*

4) One of the endpoints analyzed in several trials regarding this topic was cardiovascular death. If the authors have those data, they should report them in the manuscript.

- Response: *Attending the reviewer’s suggestion, we have reported the data regarding cardiovascular death in Table 3 (page 9): “Cardiovascular death n(%) – In hospital CR 2 (1.53) – After discharge CR 2 (1.57) – p-value 0.626”.*

5) The authors should underline the safety of a staged approach nowadays, probably related to the efficacy of modern drug eluting stents. Most of the trials included also in the recent guidelines sometimes included patients that were not treated with DES. At this regard I suggest citing the following work that underlined this concept and performed an analysis on the effect of DES in clinical outcomes in patients with STEMI and MVD: -Panuccio G, Salerno N, Rosa SD, Torella D. Timing of Complete Revascularization in Patients with STEMI and Multivessel Disease: A Systematic Review and Meta-Analysis. RCM 2023;24. doi: 10.31083/j.rcm2402058.

- Response: *Attending the reviewer’s suggestion, we have added the next paragraph in the Discussion (page 11, paragraph 1, lines 8-13): “Furthermore, our study underlines the safety of a staged approach in these patients, probably related to the efficacy of modern drug-eluting stents (DES), and in contrast to the trials included in the actual ESC Guidelines where a significant part of the patients were no treated with DES. In this regard, our findings in terms of safety are in line with a recent Meta-analysis published by Panuccio et al, demonstrating the safety of the use of DES in terms of clinical outcomes in patients with STEMI and MVD^13”^. Additionally, we have also included the next reference to the References (page 15, reference 13): “13. Panuccio G, Salerno N, Rosa SD, Torella D. Timing of Complete Revascularization in Patients with STEMI and Multivessel Disease: A Systematic Review and Meta-Analysis. Rev Cardiovasc Med. 2023;24(2), 58. doi: 10.31083/j.rcm2402058”.*

**Reviewer #2:**

Please see the following comments and questions where the manuscript is quoted followed by the question:

Introduction

‘major cardiovascular adverse events (MACE)’ – please revise to standard work sequence to match the acronym

- Response: *Attending the reviewer’s suggestion, we have revised to standard work sequence to match the acronym in the Introduction (page 3, paragraph 1, line 7): “In this particular scenario, a complete revascularization (CR) strategy of the nonculprit lesions has been associated with a reduction in major adverse cardiovascular events (MACE)^4-8^”.*

‘…(such as increasing the stress of the patients,…’ - I would consider removing this if you do not have references to support. In my opinion some patients may be stressed to wait in hospital while others may to stressed to have lesions left unmanaged at discharged. Accordingly, best to just remove this statement.

- Response: *Attending the reviewer’s comment, we have removed this statement from the Introduction (page 3, paragraph 1, line 11): “However, this non-delayed strategy may increase the risk of side effects (such as contrast induced nephropathy or bleeding)” .*

‘Thus, in this study, we aimed to examine the effect of both CR trategies on the length of stay of index hospitalization in STEMI patients with MVD.’ Since your hypothesis is also that this will be completed safely, I would consider revising to read something like this – if you agree “”Thus, in this study, we aimed to examine the effect of both CR strategies on the length of stay of index hospitalization in STEMI patients with MVD and secondarily their MACE events.””

-Response: *Attending the reviewer’s suggestion, we have added this sentence to the Introduction (page 3, paragraph 1, line 21): “ Thus, in this study, we aimed to examine the effect of both CR strategies on the length of stay of index hospitalization in STEMI patients with MVD and secondarily their MACE events”.*

Methods

‘For defining ischemia-driven revascularization, we included all PCI or CABG occurring after the baseline procedure and justified by recurrent symptoms or objective evidence of significant ischemia on provocative testing.’ Please clarify this statement. I assume that it means all revascularization after randomization and not after the index procedure? It would be atypical to count events prior to randomization.

- Response: *Attending the reviewer’s comment, we have changed this sentence in the Methods (page 5, paragraph 4, line 6): “ For defining ischemia-driven revascularization, we included all PCI or CABG occurring after randomization and justified by recurrent symptoms or objective evidence of significant ischemia on provocative testing”.*

Results

Abstract ‘At 12-month of follow-up, no differences were found in the occurrence of MACE, 7 (5.34%) patients in in-hospital CR and 4 (3.15%) in after-discharge CR strategy; (hazard ratio, 0.59; 95% confidence interval, 0.17 to 2.02; p=0.397).’

Table 10 (7.63) vs. 6 (4.72) 0.59 (0.17- 2.02)’
Results text section ‘At 12-month of follow-up, the secondary endpoint occurred in 7 of 131 patients (5.34%) in the in-hospital CR strategy and in 4 of 127 (3.15%).

The key secondary results appear different in the abstract, the results table and the results text?

- Response: *Attending the reviewer’s comment, we have corrected the error. Due to an error, in Table 3, in the secondary outcome paragraph, the total number of events were expressed incorrectly. We counted separately the patients with all cause death, myocardial infarction and ischemia-driven revascularization, whilst the patients with myocardial infarction and ischemia-driven revascularization are really the same patients. That is why in the composite of the secondary outcome the real final events are 7 (5.34%) in the in-hospital group and 4 (3.15%) in the after-discharge group, and are the same as in the Abstract and the results text. Therefore, we have changed the results in Table 3(page 9): “Secondary outcome: 7(5.34) vs. 4 (3.15) 0.59 (0.17-2.02) p=0.397”.*

Discussion

‘Although an immediate, during primary PCI, nonculprit PCI strategy has not been explored, we believe that it would not lead to a further reduction in the length of stay.’ I would revise this since there are several trials that have explored immediate culprit plus non-culprit PCI in the setting of MVD and STEMI.

- Response: *In accordance with the reviewer, there are several trials that have explored immediate culprit plus non-culprit PCI in the setting of MVD and STEMI. What we wanted to say in that sentence, was that “Although an immediate, during primary PCI, nonculprit PCI strategy has not been explored in our work…”. That is why, in order to avoid misunderstandings and attending the reviewer’s comment, we have added to the text the next sentence (page 11, paragraph 2, line 2): “Although an immediate, during primary PCI, nonculprit PCI strategy has not been explored in our work, we believe that it would not lead to a further reduction in the length of stay”.*

Additional discussion comments – the discussion is a bit repetitive that the current work is novel. Although I agree it is important and novel – this does not need to be stated more that once.

- Response: *Attending the reviewer’s comment, we have only left this initial sentence in the Discussion (page 10, paragraph 4, lines 1-3): “To the best of our knowledge, this is the first RCT to evaluate the impact of different revascularization strategies on the length of hospital stay in patients with STEMI and MVD”.*

**Journal requirements:**

1. Please ensure that your manuscript meets PLOS ONE's style requirements, including those for file naming. The PLOS ONE style templates can be found at

<https://journals.plos.org/plosone/s/file?id=wjVg/PLOSOne_formatting_sample_main_body.pdf> and

<https://journals.plos.org/plosone/s/file?id=ba62/PLOSOne_formatting_sample_title_authors_affiliations.pdf>

- Response: *We ensure that our manuscript meets PLOS ONE’s style requirements.*

2. We note that you have selected “Clinical Trial” as your article type. PLOS ONE requires that all clinical trials are registered in an appropriate registry (the WHO list of approved registries is at <https://www.who.int/clinical-trials-registry-platform/network/primary-registries>" <https://www.who.int/clinical-trials-registry-platform/network/primary-registries> and more information on trial registration is at <http://www.icmje.org/about-icmje/faqs/clinical-trials-registration/>). Please state the name of the registry and the registration number (e.g. ISRCTN or [ClinicalTrials.gov](http://ClinicalTrials.gov)) in the submission data and on the title page of your manuscript. a) Please provide the complete date range for participant recruitment and follow-up in the methods section of your manuscript. b) If you have not yet registered your trial in an appropriate registry, we now require you to do so and will need confirmation of the trial registry number before we can pass your paper to the next stage of review. Please include in the Methods section of your paper your reasons for not registering this study before enrolment of participants started. Please confirm that all related trials are registered by stating: “The authors confirm that all ongoing and related trials for this drug/intervention are registered”. Please see <http://journals.plos.org/plosone/s/submission-guidelines#loc-clinical-trials>for our policies on clinical trials.

- Response: *We have stated the name of the registry and the registration number in the title page (Page 1): “****ClinicalTrials.gov number:*** *NCT04743154”.*

3. Thank you for stating the following financial disclosure:

“The funders had no role in study design, data collection and analysis, decision to publish, or preparation of the manuscript.”

At this time, please address the following queries:

a) Please clarify the sources of funding (financial or material support) for your study. List the grants or organizations that supported your study, including funding received from your institution.

b) State what role the funders took in the study. If the funders had no role in your study, please state: “The funders had no role in study design, data collection and analysis, decision to publish, or preparation of the manuscript.”

c) If any authors received a salary from any of your funders, please state which authors and which funders.

d) If you did not receive any funding for this study, please state: “The authors received no specific funding for this work.”

Please include your amended statements within your cover letter; we will change the online submission form on your behalf.

- Response: *We have stated the following financial disclosures (page 13): “****Funding:*** *None to declare. The funders had no role in study design, data collection and analysis, decision to publish, or preparation of the manuscript. The authors received no specific funding for this work”.*

4. In your Data Availability statement, you have not specified where the minimal data set underlying the results described in your manuscript can be found. PLOS defines a study's minimal data set as the underlying data used to reach the conclusions drawn in the manuscript and any additional data required to replicate the reported study findings in their entirety. All PLOS journals require that the minimal data set be made fully available. For more information about our data policy, please see <http://journals.plos.org/plosone/s/data-availability>.

Upon re-submitting your revised manuscript, please upload your study’s minimal underlying data set as either Supporting Information files or to a stable, public repository and include the relevant URLs, DOIs, or accession numbers within your revised cover letter. For a list of acceptable repositories, please see <http://journals.plos.org/plosone/s/data-availability#loc-recommended-repositories>. Any potentially identifying patient information must be fully anonymized.

Important: If there are ethical or legal restrictions to sharing your data publicly, please explain these restrictions in detail. Please see our guidelines for more information on what we consider unacceptable restrictions to publicly sharing data: <http://journals.plos.org/plosone/s/data-availability#loc-unacceptable-data-access-restrictions>. Note that it is not acceptable for the authors to be the sole named individuals responsible for ensuring data access.

We will update your Data Availability statement to reflect the information you provide in your cover letter.

- Response:  *We specify that our study’s minimal data set underlying the results described in our manuscript has been uploaded and can be found as a Supporting Information file.*
