# Supplementary material for: In-hospital versus after-discharge complete revascularization in patients with ST segment elevation myocardial infarction and multivessel disease. REVIVA-ST trial
Source: PLoS One. 2024 May 14;19(5):e0303284. doi: 10.1371/journal.pone.0303284 (PMC11093342; doi:10.1371/journal.pone.0303284)
Supplement: Attachment — Submitted filename: Response to Reviewers.docx [file pone.0303284.s006.docx]

**RESPONSE TO REVIEWERS**

We acknowledge to the Editorial Office and Reviewers for their comments and constructive suggestions, which undoubtedly have improved the quality of this manuscript.

**Additional Editor Comments:**

*The authors have addressed most of the comments but there is still much room for improvements.
I kindly recommend the authors to revise the manuscript according to reviewers' comments and to update the introduction and discussion on the topic by citing most recent trials, international guidelines and reviews on the topic (such as PMID: 33036712; PMID: 37634190; PMID: 37622654; PMID: 38343368).*

**Response:** Following the editor’s comments, we updated the introduction and discussion on the topic by citing the recommended recent trials, international guidelines and reviews on the topic.

- Discussion, page 11: “Multiple studies have evaluated the efficacy of a CR in STEMI patients with MVD, however, even today, the optimal time to perform it still remains a matter of debate^14^. In this regard, our study demonstrates…”
- References, page 16: “14. Ilardi F, Ferrone M, Avvedimento M, Servillo G, Gargiulo G. Complete Revascularization in Acute and Chronic Coronary Syndrome. Cardiol Clin. 2020;38(4):491-505.”
- Discussion, pages 11 and 12: “Although an immediate, during primary PCI, nonculprit PCI strategy has not been explored in our work, we believe that it would not lead to a further reduction in the length of stay. Recently, the Multivessel Immediate versus Staged Revascularization in Acute Myocardial Infarction (MULTISTARS AMI) trial, demonstrated that immediate multivessel PCI at the time of primary PCI was noninferior to staged multivessel PCI in patients with STEMI and MVD, reporting a median hospital stay of 4 days (3-6) in the immediate CR strategy, similar to the data obtained in the delayed CR group of our work^17^. Likewise, Politi et al observed no differences in this setting between STEMI patients revascularized during primary PCI or in a staged procedure^17^. By contrast,”
- Discussion, page 13: ¨ Accordingly, the MULTISTARS trial also presented an increase in 12-month MACE, mostly due to nonfatal MI and unplanned ischemia-driven revascularization, in the staged CR strategy. It is important to highlight that, no apparent differences in the percentage of patients with spontaneous MI were observed between the trial groups. Furthermore, the difference observed was predominantly due to an elevated rate of procedure-related MI, which is a really difficult MACE to quantify in an immediate strategy.”
- References, page 16: “16. Stähli BE, Varbella F, Linke A, Schwarz B, Felix SB, Seiffert M, et al. Timing of Complete Revascularization with Multivessel PCI for Myocardial Infarction. N Engl J Med. 2023;389(15):1368-1379.”
- Introduction, page 3: “but always within the first 45 days. Therefore, the recent 2023 ESC Guidelines for the management of acute coronary syndromes, recommend a CR either during the index PCI procedure or within 45 days (class of recommendation I, level of evidence A)^10^. Since the optimal timing of revascularization (immediate during the index procedure vs. staged within 45 days) has not yet been evaluated in adequate randomized clinical trials (RCTs), no recommendation has been endorsed in this sense in the guidelines.¨
- References, page 16: “10. Byrne RA, Rossello X, Coughlan JJ, Barbato E, Berry C, Chieffo A, et al. 2023 ESC Guidelines for the management of acute coronary syndromes. Eur Heart J. 2023; 44(38):3720–3826.”
- Introduction, page 3: “Both of these strategies have pros and cons^11^, and therefore, the optimal timing for CR still today remains a subject of debate.”
- References, page 16: “11. Kastrati A, Kessler T, Rinaldi R, Brugaletta S. Complete revascularisation should be immediate in STEMI: pros and cons. EuroIntervention. 2024;20(3):e171-e173.”

**Review Comments to the Author**

**Reviewer #1:** *The colleagues revised the manuscript according to our revision, and the quality of the manuscript is improved.
However, there are other issue to solve:*

*-There are still typos in the text. In the discussion section for example there is still a “de”. Please be sure to revise all typos.*

*“However, in this study only 40% of patients presented with STEMI and the staged PCI could be performed within 6 weeks after de index PCI, both during the hospitalization or after discharge. Moreover, we do not actually know whether the increase in hospital stay is a consequence of a longer index admission or secondary to the deferred procedure”.*

**Response:** In accordance with the reviewer’s comment, we have revised all typos in the text.

- Discussion, page 11: “However, in this study only 40% of patients presented with STEMI and the staged PCI could be performed within 6 weeks after the index PCI, both during the hospitalization or after discharge”.

*-In the sentence “The current guidelines recommendations on revascularization of non-culprit lesions in STEMI, is based on the data from RCTs that evaluate the benefit of a CR over culprit vessel only PCI. However, as far as we know, there are actually no RCTs focusing on the efficacy of different CR strategies in STEMI patients. The BIOVASC trial^15^ showed that an inmediante CR was non-inferior to a staged CR for the combined endpoint (all cause mortality, MI, unplanned ischaemia- driven revascularization or cerebrovascular event) at 12 months of follow-up. However, this trial was performed in a cohort of patients with ACS, where 60% were non-STEMI patients.”*

*First of all, the sentence “The current guidelines recommendations on revascularization of non-culprit lesions in STEMI, is based on the data from RCTs that evaluate the benefit of a CR over culprit vessel only PCI” must be revised with “The current guidelines recommendations on revascularization of non-culprit lesions in STEMI , are based on the data from RCTs that evaluate the benefit of a CR over culprit vessel only PCI.” Also, there is another typo in the text (“inmediante”).*

*Finally, the sentence “However, as far as we know, there are actually no RCTs focusing on the efficacy of different CR strategies in STEMI patients.” is not true. A subgroup analysis of the CVLPRIT trial as well as Corpus et al (Corpus RA, House JA, Marso SP, et al. Multivessel percutaneous coronary intervention in patients with multivessel disease and acute myocardial infarction. Am Heart J. 2004;148(3):493-500. doi:10.1016/j.ahj.2004.03.051) compared different strategies of revascularization. Please revise.*

**Response:** Following the reviewer’s suggestion, we have modified the following sentences in the text.

- Discussion, page 12: “The current guidelines recommendations on revascularization of nonculprit lesions in STEMI^9^, are based on the data from RCTs that evaluate the benefit of a CR over culprit vessel only PCI. However, as far as we know, actually exists scarce scientific evidence that focuses on the evaluation of the optimal-timing of the different CR strategies in STEMI patients with MVD. The BIOVASC trial^16^ showed that an immediate CR was non-inferior to a staged CR for the combined endpoint (all cause mortality, MI, unplanned ischaemia-driven revascularization or cerebrovascular event) at 12 months of follow-up. However, this trial was performed in a cohort of patients with ACS, where 60% were non-STEMI patients.”

**Reviewer #3:** *The Authors submitted the manuscript "In-hospital versus after-discharge complete revascularization in patients with ST segment elevation myocardial infarction and multivessel disease. REVIVA-ST trial."*

*A RCT comparing in-hospital vs discharge coronary revascularization in patients with STEMI.*

*Some comments:*

1) *Please refer to the last ESC ACS guidelines of 2023.*

**Response:** Following the reviewer’s comment, we have added the recent 2023 ESC ACS Guidelines in the references and we have modified the text in order to refer to these guidelines.

- Introduction, page 3: “However, the optimal timing to perform this “protective” PCI remains uncertain. The 2017 European Society of Cardiology (ESC) STEMI Guidelines recommended a CR strategy preferably during index admission before discharge^9^. The main rationale for this recommendation was due to the fact that all trials available until then had performed MVD PCI within that specific time frame. However, this non-delayed strategy may increase the risk of side effects (such as contrast induced nephropathy or bleeding) and logistic constrains that ultimately increase the length of index hospitalization. Recently, the COMPLETE trial, demonstrated a 3-year reduction in the combined endpoint of death or MI with staged PCI (performed within 45 days of STEMI) of the non-infarct related artery compared with culprit-vessel only PCI^8^. These benefits were consistent, irrespective of the timing of the non-infarct artery PCI, whether it was performed during admission or after discharge, but always within the first 45 days. Therefore, the recent 2023 ESC Guidelines for the management of acute coronary syndromes, recommend a CR either during the index PCI procedure or within 45 days (class of recommendation I, level of evidence A)^10^. Since the optimal timing of revascularization (immediate during index hospitalization vs. staged within 45 days) has not yet been evaluated in adequate randomized clinical trials (RCTs), no recommendation has been endorsed in this sense in these guidelines. Along with these lines of thoughts, we speculate that…”
- References, page 16: “10. Byrne RA, Rossello X, Coughlan JJ, Barbato E, Berry C, Chieffo A, et al. 2023 ESC Guidelines for the management of acute coronary syndromes. Eur Heart J. 2023; 44(38):3720–3826.”

2) *The Authors should add more info on the identification of events of interest during follow-up. How were identified? Any remote follow-up beyond the outpatient visits?*

**Response:** In accordance with the reviewer’s comment, we have to clarify that the identification of events of interest during follow-up were not only identified during the scheduled outpatient visits, but also by interviewing the hospital electronic database at 1 year (±14 days) after index revascularization. Therefore, we have modified the following paragraph:

- Methods, page 6: “Follow-up was conducted during outpatient clinic visits scheduled at 30 days, 6 months and 12 months after primary revascularization, and also by means of hospital electronic database.”

3) *What about contrast medium? Any difference between group? The Authors should provide info on eGFR/creatinine at baseline and 12-month follow-up to have further info on safety profile between the 2 revascularization strategies.*

**Response:** According to the reviewer`s suggestion, we added the volume of contrast used, as well as the creatinine at baseline and at 12-month follow-up in table 1 and 2.

- Results, Table 1, page 8:

| Creatinine at baseline, mg/dl | 1.5 ± 0.55 | 0.98 ± 0.75 | 0.862 |
| --- | --- | --- | --- |

- Results, Table 2, page 8 and 9:

| Volume of contrast used, ml | 110 (90-115) | 105 (72-108) | 0.746 |
| --- | --- | --- | --- |
| Creatinine at 12-month follow-up, mg/dl | 1.48 ± 0.43 | 0.96 ± 0.61 | 0.634 |

4) The Authors should include peri-procedural bleeding events. Did they find any differences between groups?

**Response:** Following the reviewer`s suggestion, we added periprocedural major bleedings in table 2, observing no differences between both groups. Therefore, we added the following sentence to the text:

- Results, page 7: “No differences were observed between the two group strategies in terms of peri-procedural major bleeding (BARC ≥ 3) or vascular access complications.”
- Results, Table 2, page 9:

| Peri-procedural major bleeding, n (%) | 2 (1.53) | 0 (0) | 0.161 |
| --- | --- | --- | --- |

5) I suggest to add info on vascular access complications for completeness of safety profile comparing the two strategies.

**Response:** In accordance with the reviewer`s comment, we added vascular access complications in table 2, observing no statistical differences between both group strategies. Therefore, we added the following sentence to the text:

- Results, page 7: “No differences were observed between the two group strategies in terms of peri-procedural major bleeding (BARC ≥ 3) or vascular access complications.”
- Results, Table 2, page 9:

| Vascular access complication, n (%) | 1 (0.76) | 0 (0) | 0.320 |
| --- | --- | --- | --- |

**Reviewer #4:** *The manuscript addresses an interesting topic. The employed statistical methods are rather sound, though standard. A few more details are required to ensure the reliability of the results. Comments follow.*

*1. Strict assumptions must be met to get reliable inference when t-tests are employed. Please, provide evidence that all the assumptions, from the normality to homoskedasticity, etc. are met.*

**Response:** You are right. The results from the Shapiro-Wilk test for length of stays indicate that the data do not follow a normal distribution, as shown below.

W: The W value is 0.85360. This is the test statistic and reflects how well the data fit a normal distribution. Values closer to 1 indicate a better fit to the normal distribution.

Prob>z: The p-value is 0.00000, which is less than the common significance threshold of 0.05 (or any other threshold you might be using, such as 0.01). This means there is sufficient statistical evidence to reject the null hypothesis that the data are normally distributed.

In the revised version of the text, we reanalyze the between-treatment difference for length of stay using the Mann-Whitney test. This analysis revealed the median length of stays was lower in the active arm (as shown below). The methods section were changed accordingly.

- Methods, page 6: All patients who underwent randomization were included in the analysis according to the treatment group to which they were assigned, regardless of the CR strategy they actually received (intention-to-treat principle). Because the length of stay did not meet the criteria for a parametric distribution (assessed by the Shapiro-Wilk test), the primary endpoint (length of stay) between treatment arms was analyzed by the Mann-Whitney test.

Accordingly, the results section was changed. Basically, the meaning are in the same line. Patients in the active arm showed a lower length of stay.

- Results, page 9: Clinical outcomes are summarized in Table 3. The median length of hospital stay (p25%-p75%) was significantly higher in the in-hospital CR strategy, when compared with the after-discharge CR group [7 days (5-9) versus 4 days (3-5); p<0.001]. There were no differences between both strategies in terms of length of stay in the median coronary care unit (CCU) [2 days (2-3) vs 2 days (2-3), p=0.631], respectively. The median length of stay after a non-culprit revascularization procedure in the after-discharge strategy was inferior to 24 hours in all patients [0.38 days (0.25-0.81)].

*2.As is the case for a linear or generalized linear model, it is desirable to determine whether a fitted Cox regression model adequately describes the data. I will briefly consider three kinds of diagnostics: for violation of the assumption of proportional hazards; for influential data; and for nonlinearity in the relationship between the log hazard and the covariates. All of these diagnostics use the residuals method, which requires the calculation of several kinds of residuals (along with some quantities that are not normally thought of as residuals). Tests and graphical diagnostics for proportional hazards may be based on the scaled Schoenfeld residuals. Nonlinearity – that is, an incorrectly specified functional form in the parametric part of the model – is a potential problem in Cox regression. The martingale residuals may be plotted against covariates to detect nonlinearity, and may also be used to form component-plus-residual (or partial-residual) plots, again in the manner of linear and generalized linear models.*

**Response:** Thanks for your input. Following your suggestion, we tested the proportionality assumption of the Cox regression model using Schoenfeld residuals.

Revcompext (type of revascularization strategy)

As you may appreciate in the output (see below) the hazard ratios between groups are constant over time. The test for this assumption, based on the rank of analysis time and focusing on the **revcompext** variable (our exposure), gives a rho value of -0.11517 and a chi-square value of 0.15 with 1 degree of freedom, resulting in a p-value of 0.7028. This indicates no evidence against the proportional-hazards assumption for the **revcompext** variable, as the p-value is well above the conventional threshold of 0.05. The global test, which assesses the overall model, also supports the proportional-hazards assumption with a p-value of 0.7028.

It was included into the revised version of the manuscript.

- Methods, page 6: “Proportional-hazard assumption of the survival model was verified by using Schoenfeld residuals (p-value: 0.703)”.

*3. I strongly suggest to provide more information on the relevant variables in the survival model. Maybe, a lasso approach could be used to select the relevant variables, dropping redundant and non-informative variables.*

**Response:** We want to clarify that it is a randomized clinical trial. Estimated were unadjusted in all cases. In a randomized clinical trial, randomization serves as a powerful tool designed to balance both known and unknown covariates across treatment groups, aiming to minimize bias and establish a fair comparison of the treatments. While adjusting for covariates is a common practice in observational study analyses to control for confounding bias, there are several reasons why such adjustment might not be necessary or even desirable in a randomized clinical trial. Additionally, we only registered 11 adverse clinical events in the survival analysis. The inclusion of covariates will also overfit the model. We clarify in the revised test that estimates are unadjusted.

- Methods, page 6: “Estimates of risk were also assessed by using Cox regression analysis, and differences expressed as unadjusted hazard ratios (HR) and 95% confidence interval (CI)”.
